# Case report: Uncommon immune-mediated skin disease involving systemic disorders in dogs

**DOI:** 10.3389/fvets.2022.915775

**Published:** 2022-09-02

**Authors:** Soomin Kim, Yoonji Kim, Ha-Jung Kim

**Affiliations:** ^1^Department of Veterinary Internal Medicine, College of Veterinary Medicine, Chonnam National University, Gwangju, South Korea; ^2^BK21 Project Team, College of Veterinary Medicine, Chonnam National University, Gwangju, South Korea

**Keywords:** immune mediated skin diseases, adverse drug reaction, *Pemphigus foliaceus*, dog, immune suppressive agents

## Abstract

Case 1, a 6-year-old, spayed female Pug, presented with severe systemic urticaria, edema, and erythema. The dog had received a famotidine injection as a treatment for repeated vomiting in another hospital. On physical examination, hyperthermia was observed. Moderate pancytopenia, hypoalbuminemia, and increased CRP and D-dimer were also observed in blood tests. Hyposthenuric proteinuria, pulmonary interstitial infiltration, and hepatomegaly were found in other tests. In the histology of the skin, dermal edema and infiltration of inflammatory cells were observed. Therefore, she was diagnosed with acute systemic hypersensitivity. Case 2, a 13-month-old, neutered male Pembroke welsh corgi, presented with severe and patchy systemic ulcerative skin lesions. The dog had a history of soft feces and pain around the anus 2 days before. Thrombocytopenia, and increased CRP and D-dimer were observed in blood tests. In histology, epidermal necrolysis, separation of the epidermis and dermis, and infiltration of inflammatory cells were observed. Therefore, he was diagnosed with an immune-mediated disease with necrolysis dermatitis. Case 3, a 12-year-old, spayed female Pomeranian, presented with severe systemic alopecia, pustule, and crust on the skin. The dog had received an infection treatment from a local hospital. Severe regenerative anemia (hematocrit 15.3%, negative saline agglutination test, negative slide agglutination test, negative Coomb's test, prominent spherocytes) elevated liver enzymes, and increased CRP and D-dimer were observed in blood tests. On histopathology of the skin, pustules, acantholytic cells, and inflammatory cells were observed in the keratin layer of the epithelium. Therefore, she was diagnosed with *Pemphigus foliaceus* concurrent with immune-mediated hemolytic anemia. The 3 cases were diagnosed with fatal immune-mediated skin disease concurrently with hematological and systemic abnormalities. All the cases were treated with immune-suppressive drugs, prednisolone, and cyclosporine. In cases 2 and 3, the dogs also received human intravenous immunoglobulin as an immune modulator. The treatment was successful with significant improvements in all the 3 cases.

## Introduction

Immune-mediated skin diseases are rare but challenging diseases in small animals (1). They are reported to comprise 1.4% of all canine skin diseases and 1.3% of all feline skin diseases (2). Sometimes the condition spreads and targets multiple organs including systemic vessels in dogs. Such cases are life-threatening and their management could be challenging.

Adverse drug reaction (ADR) is an unintended and harmful response to a drug (3, 4). In humans, clinical signs vary from skin changes to cardiovascular changes or bronchospasm (5). In dogs, ADRs have been reported to present with skin changes and thrombocytopenia (6–8). Cutaneous ADR is an adverse drug reaction that appears on the skin first (2), and the lesions imitate any other skin disease (2, 9, 10). Immune-mediated hypersensitivity can be classified as an ADR and its phenotype such as anaphylaxis, urticaria, edema, and vasculitis can occur in both humans and dogs (9, 11). Skin lesions such as papules, pustules, and angioedema are also included (12).

Famotidine and antibiotics like quinolones and sulfonamide can cause hypersensitivity and anaphylaxis in humans (12–14). Foods such as beef, dairy products, eggs, wheat, and nuts are also known causes of anaphylactic reactions in humans and dogs (15, 16).

*Pemphigus foliaceus* (PF) is the most common autoimmune disease in dogs, that occurs in the subcorneal region of the epidermis (17, 18) and presents as skin lesions including pustules, crusts, and alopecia (19). With *Pemphigus*, systemic symptoms like lymphadenomegaly, limb edema, fever, anorexia, and depression can also occur (20). Further, *Pemphigus* can be accompanied by immune-mediated hemolytic anemia (IMHA) in humans and immune-mediated thrombocytopenia in very rare cases, in dogs (21, 22). The present case series describe the successful management of rare immune-mediated skin diseases that occurred concurrently with fatal systemic abnormalities using immune-suppressive drugs in dogs.

## Case presentations

Case 1) A 6-year-old, spayed female Pug presented with urticaria, severe systemic epidermal edema, erythema, and dehydration. The dog had received a famotidine (1 mg/kg, once) injection due to gastrointestinal symptoms, vomiting. After that, erythema and petechiae were observed on the whole body. A few days later, the dog had received the additional dose (0.5 mg/kg, bid) of famotidine injection. Then, anorexia and weakness were observed along with severe generalized skin lesions.

**Figure 1:**
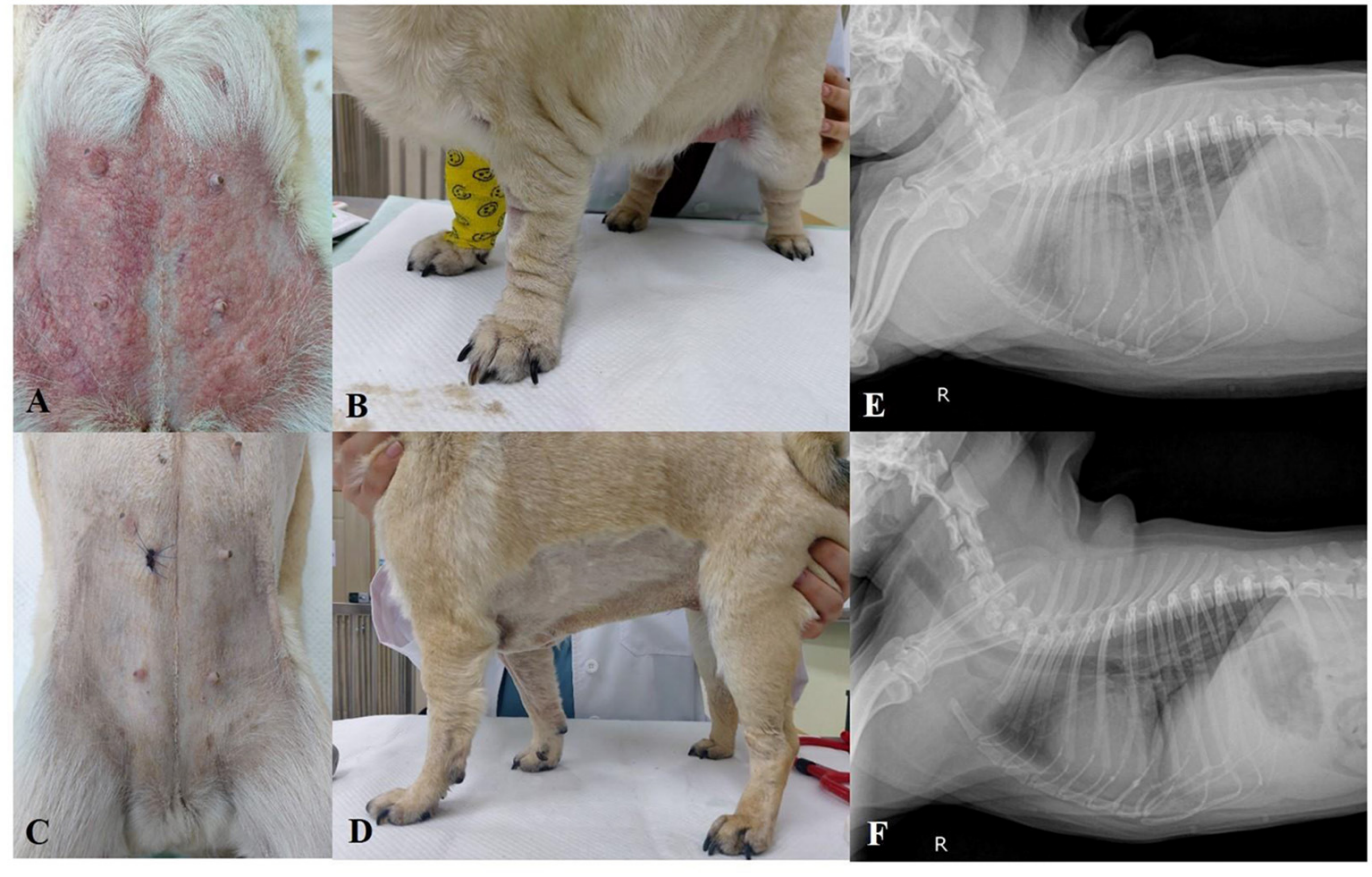
Photographs of the skin condition of Case 1. **(A,B)** Severe systemic erythema, edema, and urticaria at presentation. **(C,D)** One week after treatment initiation, erythema and edema disappeared. **(E)** Pulmonary interstitial infiltration and right-side cardiomegaly at the presentation on X-ray. **(F)** One week later, after treatment initiation, the infiltration of the lung disappeared.

Examinations results showed systemic hypotension (78 mmHg), hyperthermia (about 40 °C), delayed skin turgor, and labored breathing were found. Severe and generalized erythema, edema, and urticaria were also presented (Figures 1A,B). Complete blood cell count showed severe pancytopenia (non-regenerative anemia, neutropenia, and thrombocytopenia). Auto-agglutination, spherocytes, and infectious agents were not found in the blood smear. Coombs test was negative. Decreased total protein and albumin, increased C-reactive protein (CRP), hypertriglyceridemia in serum chemistry, prolonged activated partial thromboplastin time (aPTT), and elevated D-dimer were found in the coagulation test (Supplementary Table 1). Pancreatitis was ruled out by the measurement of serum canine specific lipase concentration (Eurolyser Lipase Test Kit, Eurolyser Diagnostica GmbH, Salzburg, Austria). An X-ray showed right side cardiomegaly, pulmonary infiltration, moderate hepatomegaly (Figure 1E) and moderated dilation of the caudal vena cava and hepatic veins were noted on ultrasonography. Although urea nitrogen and creatine were in normal range, hyposthenuria and proteinuria were noted in urinalysis, therefore, kidney function failure was suspected. As the hepatic congestion was suspected on ultrasonography, bile acid test was performed. In the result, pre-prandial and post-prandial bile acid were over 25 μmol/L and therefore liver function failure was confirmed.

In histopathology of the abdominal skin, dermal edema, and infiltration of inflammatory cells such as neutrophils, mast cells, and macrophages in the dermis with edema were found (**Figures 4A,B**). Therefore, the skin lesions were histopathologically diagnosed as acute hypersensitivity allergy. Based on all the examination results the dog was finally diagnosed with drug hypersensitivity reaction (DHR) with multiple organ function failure and famotidine was suspected as the causative drug. Furosemide (Lasix®, Handok, Seoul, Korea; 1 mg/kg, PO, bid) for pulmonary and systemic edema and congestion, rivaroxaban (Xarelto®, Bayel Korea, Seoul, Korea; 0.5 mg/kg, PO, sid) for hypercoagulation, and immunosuppressive dose of prednisolone (Solondo®, Yuhan, Seoul, Korea; 2 mg/kg, PO, bid) and cyclosporine (Cipol-N®, Chongkundang, Seoul, Korea; 7 mg/kg, PO, bid) were prescribed.

After 1 week, the edema, erythema, and urticaria disappeared (Figures 1C,D). Laboratory examinations showed that the abnormalities were significantly improved (Supplementary Table 1). Proteinuria and pulmonary infiltration also disappeared (Figure 1F). The dog went back to the local hospital.

Case 2) A 13-month-old, neutered male Pembroke Welsh Corgi presented with severe systemic ulcerative skin lesions. The dog had shown soft feces after having a new snack 2 days ago, and the owner had also seen patchy ulcerative skin lesions around the hip. The lesions were spreading to the neck and flank. The dog showed systemic weakness and anorexia.

**Figure 2:**
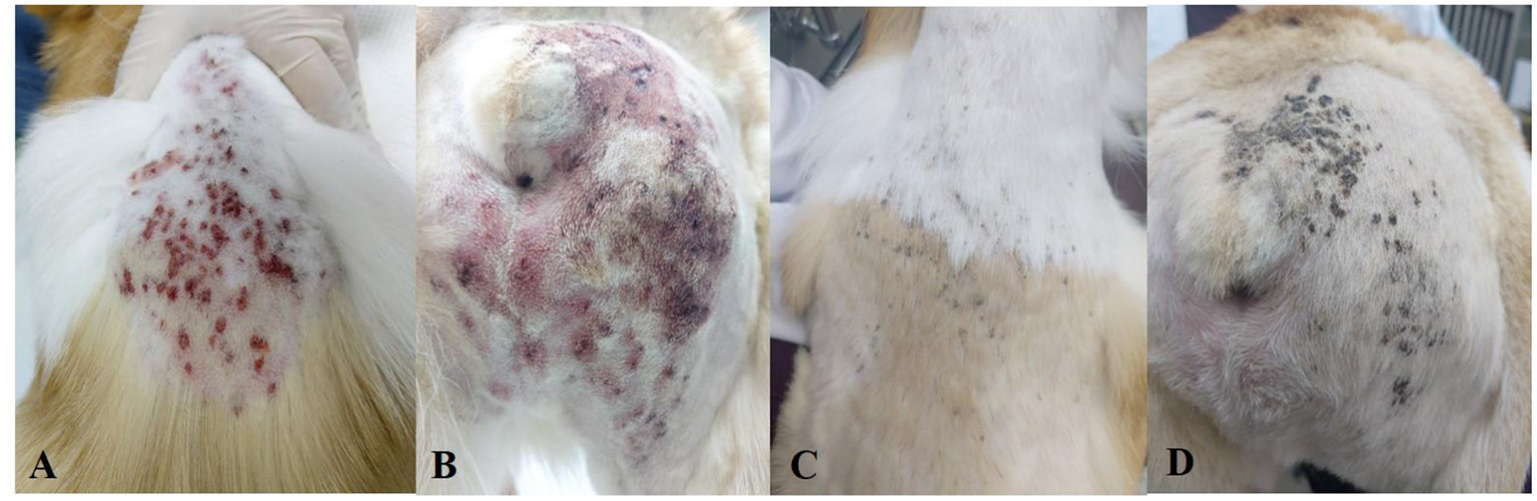
Photographs of the skin condition of Case 2. **(A,B)** Systemic ulcerative patchy lesions at presentation. **(C,D)** 10 days later, after treatment initiation, the lesions were mostly improved.

Physical examinations showed hyperthermia (about 40 °C), generalized patchy ulcerative lesions, and erythema around the skin lesions (Figures 2A,B). Complete blood cell count showed thrombocytopenia. Elevated alkaline phosphatase (ALKP) and CRP were found in serum chemistry. Prolonged aPTT and elevated D-dimer were identified in the coagulation test (Supplementary Table 1). Cytology of skin lesion impressions showed numerous sterile inflammatory cells (neutrophils and macrophages). The x-ray did not show specific findings except for mild splenomegaly. In histopathology of the dorsal skin lesions, dermis and epidermis were separated and infiltrations of inflammatory cells such as neutrophils, macrophages, and lymphocytes were found on the interface sites (**Figures 4C,D**). Based on these results, the skin lesions were diagnosed with immune-mediated skin disease induced by an unknown cause (new snack suspected). The patient received an immunosuppressive dose of prednisolone (Solondo®, Yuhan, Seoul, Korea; 2 mg/kg PO bid), cyclosporine (Cipol-N®, Chongkundang, Seoul, Korea; 7 mg/kg PO bid), and human intravenous immunoglobulin (hIVIG) (Liv -Gamma, SK Plasma, Seongnam, Korea; 0.5 g/kg, IV, 2 days in a row).

After 10 days, the patient's appetite and vitality, and the skin lesions were significantly improved (Figures 2C,D). The laboratory findings showed that the abnormalities were markedly improved (Supplementary Table 1). The prednisolone was tapered (up to 1 mg/kg PO bid) and the patient came back to the local hospital.

Case 3) A 12-year-old, spayed female Pomeranian presented with severe systemic alopecia, pruritus, pustule, and crust. About 2 months ago, she had discharge from the general skin and mild alopecia. The patient did not respond to antibiotics (amoxicillin, doxycycline, and gentamicin), antifungal agents (clotrimazole), and prednisolone (0.5 mg/kg PO bid) in the local hospital. The lesions were spreading to the whole body.

**Figure 3:**
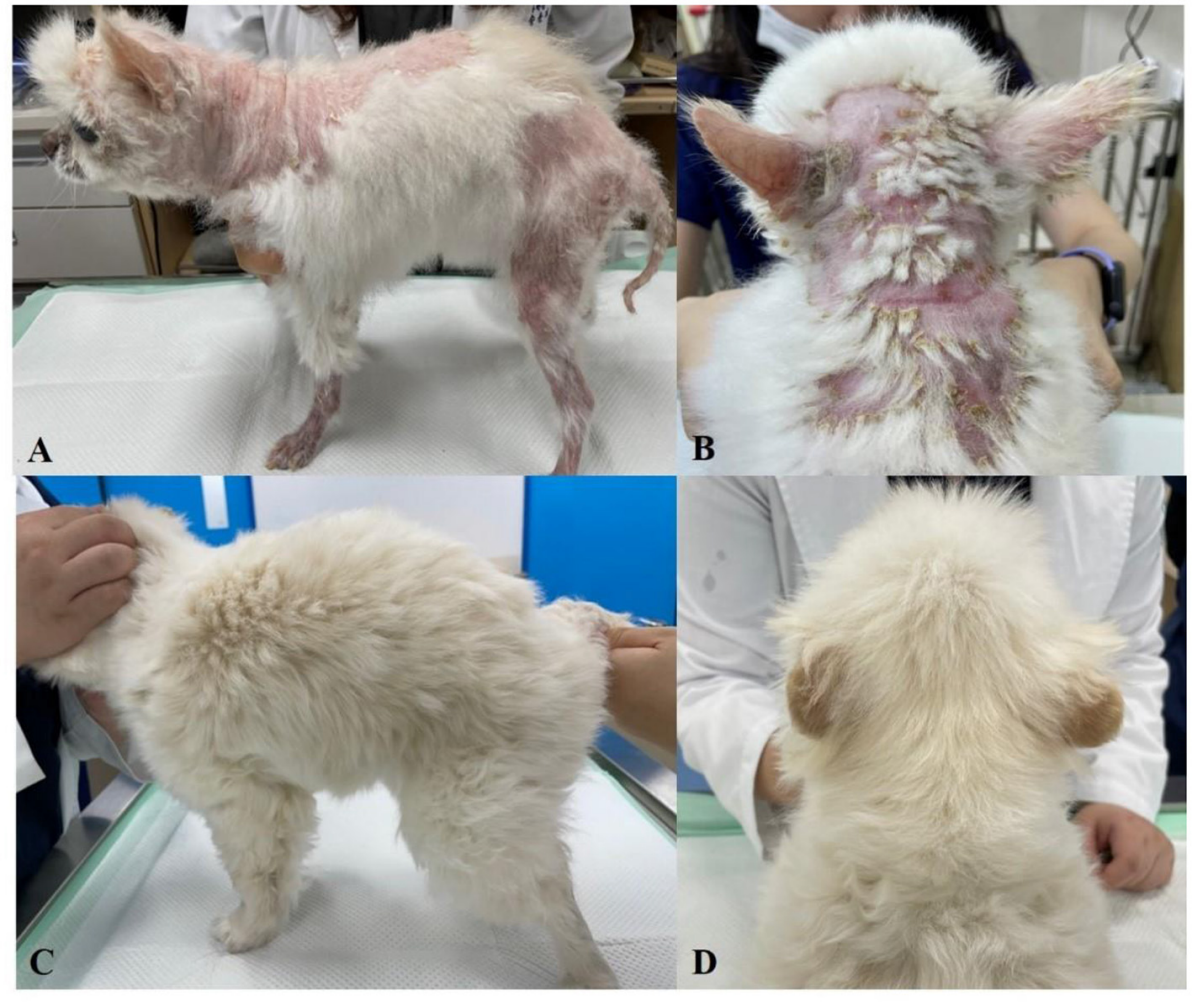
Photographs of the skin condition of Case 3. **(A,B)** Systemic alopecia and crusts at presentation. **(C,D)** 10 months later, after treatment, alopecia was significantly improved and the crusts disappeared.

**Figure 4:**
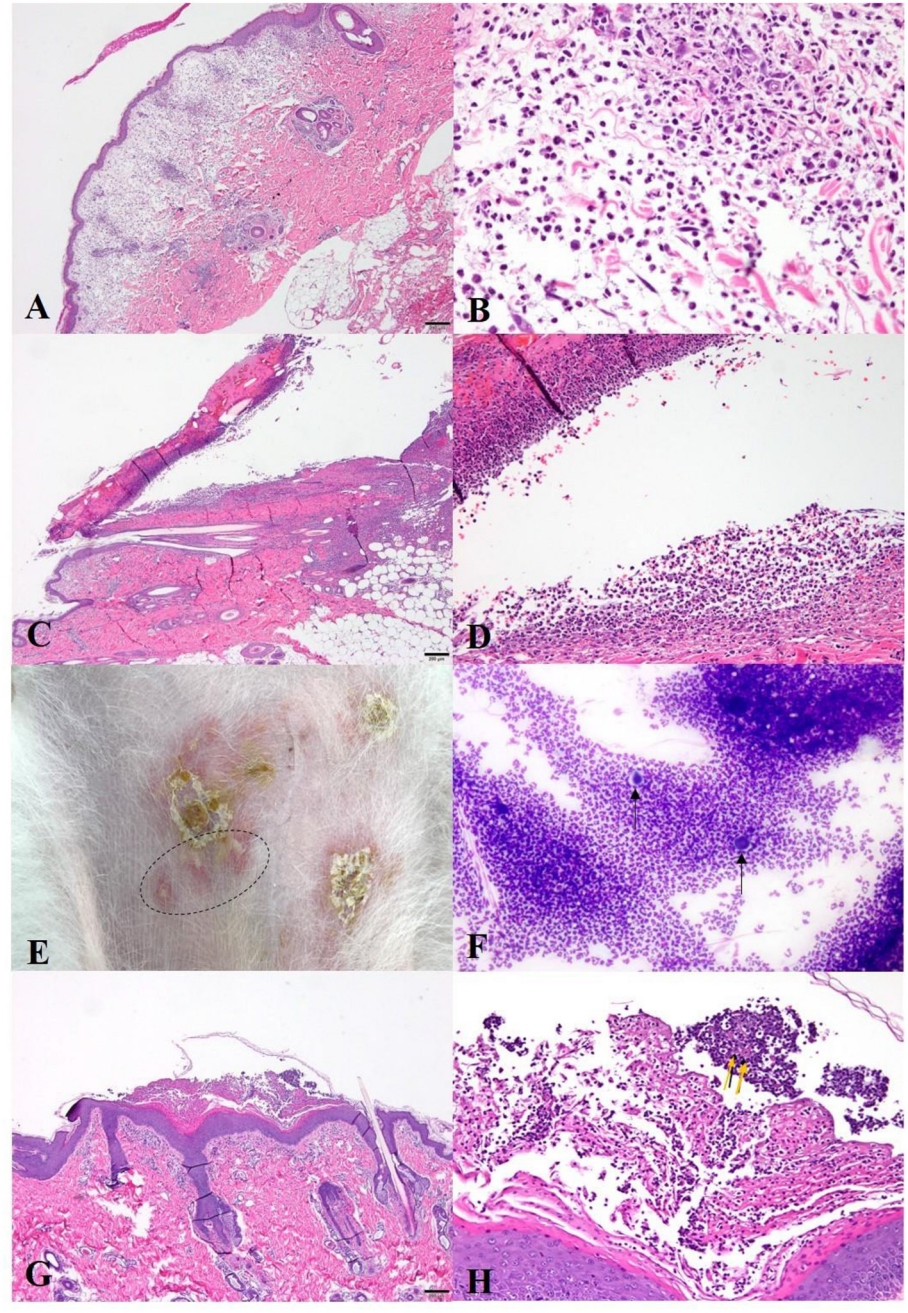
Histopathology (H&E staining) and cytology (Diff quick staining) of the [**(A,B**): Case 1]. **(A)** Marked edema with diffuse inflammation in the dermis(40x); **(B)** High magnitude of **(A)**. Inflammatory cells (neutrophils, mast cells, and macrophages) are infiltrated in the edematous dermis (400x); [**(C,D)**: Case 2] **(A)** Epidermal necrosis and separation of the epidermis and dermis (40x); **(D)** Infiltration of the inflammatory cells (neutrophils, macrophages) in separated interface sites (200x); [**(E–H)**: Case 3] **(E)** Crusts and intact pustules (dot circle) on the abdomen; **(F)** Acantholytic cells (black arrows) and numerous neutrophils on the cytology of the pustules (400x); **(G)** Pustules in the hyperplastic stratum corneum in histopathology (40x); **(H)** Acantholytic cells (yellow arrows) that are surrounded by inflammatory cells (neutrophils, macrophages) in pustule (200x).

On the physical examinations, severe generalized alopecia, crusts, and erythema were identified (Figures 3A,B). Some pustules and epidermal collarettes were also noted on the abdomen (Figure 4E). Complete blood cell counts showed severe normocytic normochromic regenerative anemia (hematocrit 15.3%) and negative on Coombs' test with no direct and indirect agglutinations. Stress leukograms were also observed. Some spherocytes were noted in the blood smear. Moderately elevated liver enzymes (ALKP, GGT) and increased CRP were found in the serum chemistry test (Supplementary Table 1).

Skin examination including Wood's lamp, skin scraping, and trichogram, did not show remarkable findings. Meanwhile, there were numerous acantholytic cells surrounding inflammatory cells (neutrophils, macrophages) in the pustule on cytology (Figure 4F). Histopathology showed pustules containing numerous acantholytic cells in the stratum corneum similar to those observed in cytology (Figures 4G,H). Based on the results, the patient was diagnosed with *Pemphigus foliaceus* concurrent with IMHA which showed no agglutination due to previous steroids administration. The patient received a blood transfusion and then was started with immune suppressants, prednisolone (Solondo®, Yuhan, Seoul, Korea; 2 mg/kg PO bid), cyclosporine (Cipol-N®, Chongkundang, Seoul, Korea; 7 mg/kg PO bid). Then hIVIG (Liv -Gamma, SK Plasma, Seongnam, Korea; 0.5 g/kg IV, for 2 days in a row) was also used for the treatment of acute immune-suppressive effects.

The prednisolone dose was tapered for over a month. Anemia was resolved (HCT 39.0%) (Supplementary Table 1). Generalized crust and pustules disappeared after a month, and the alopecia gradually improved. The patient showed ocular problems due to the steroid, so prednisolone was replaced by chlorambucil (Leukeran^TM^, Samil Pharm, Seoul, Korea; 2 mg/kg, PO, sid). The dog was well managed by immunosuppressants for 10 months (Figures 3C,D).

## Discussion

This report describes the successful management of three cases of fatal immune-mediated skin diseases. They were diagnosed as immune-mediated skin diseases relating to CADR, *Pemphigus foliaceus*, and unknown etiology. The cases showed severe systemic and skin signs. High doses of immunosuppressive drugs and supportive management were successfully applied.

Sometimes drug reaction is mainly presented with immune mediated skin problems in dogs. Immune-related drug reactions can be classified as, (1) exanthematous drug eruptions (hypersensitivity reaction type IV-T lymphocyte-cytotoxic–mediated): reappears in 1 to 3 days by subsequent exposure; (2) urticaria, angioedema, anaphylaxis (type 1 IgE–mediated reactions): emerges within minutes to hours of exposure to the offending agent and may occur in combination or individually; (3) allergic contact dermatitis (type IV- T lymphocyte-macrophage–mediated): usually occurs within 48 to 72 h of contact with the allergen including various poison agents; (4) drug hypersensitivity syndrome (type IV- T lymphocyte-eosinophil–mediated): a drug-induced maculopapular eruption accompanied by eosinophilia with fever, facial edema, and organ dysfunction (hepatic, renal, or hematologic); fixed drug eruptions; erythema multiforme, and serum sickness–like reactions (23).

Case 1 may be related to “urticaria, angioedema, anaphylaxis” and is included in second classification of above reference (23). Angioedema is characterized by a localized, self-limiting, and transient subcutaneous or submucosal swelling, which can present with or without episodes of urticarial (24). The systemic skin swelling may be due to increased vascular permeability of small vessels in dermis. The case also showed systemic signs including edematous with congestive cardio-pulmonary problems which are very rare in that skin disease in dogs. The congestive heart and systemic conditions could be related to increased vascular permeability by hypersensitivity reaction.

There are several causes of angioedema and urticaria such as food, drug, or insect in dogs (25). The dog had no certain etiology relating hypersensitivity and only previous using a drug, famotidine was suspected. It is not reported that adverse drug reactions are induced by famotidine in veterinary medicine. Notably, in humans, there is a previous study reported H_2_-receptor antagonists' adverse reactions (26). In the report, dermatological reactions, such as urticaria, pruritus, erythema, and alopecia constituted 35% of the H_2_-receptor antagonists ADRs (27). The majority of ADRs were caused by cimetidine and ranitidine (26). Famotidine ADRs are very rare with only 2 cases of ADRs that affected the skin and a case report of famotidine-induced anaphylaxis in humans reported so far (14). The patient showed dyspnea, seizure-like activities, and became comatose after intravenous injection of cefazedone and famotidine (14). There was no report about famotidine-induced cutaneous ADR accompanied by other organs' abnormalities in humans and dogs.

Food, such as cow milk, egg, shrimp, and wheat, have been reported to induce urticaria and anaphylaxis in children (28). In the dogs, probable walnut-induced anaphylaxis has been reported (15). The dog had showed acute diarrhea, vomiting, respiratory distress, and skin swellings (15). The dog in case 2 had a history of eating a new snack before the symptoms appeared. There was no history of other dietary or environmental changes except. The skin lesions may be associated with food hypersensitivity to the new snack, but unfortunately we could not confirm the information on the snack's ingredients.

On histopathology, the case 2 reveals a lichenoid interface dermatitis which is possibly associated with systemic lupus erythematosus (SLE). For the diagnosis of probable SLE, fulfillment of at least four criteria is needed (29). The criteria include erythema, discoid rash, photosensitivity, oral ulcers, arthritis, serositis, renal disorders, neurologic disorders, hematologic disorders, immunologic disorders, and antinuclear antibodies (29). Although we did not perform immunologic tests, only two abnormalities of skin and hematologic finding (thrombocytopenia) are included.

Food hypersensitivity (allergy) is included in the “adverse food reactions” which also include non-immune mediated reactions (30). IgE-mediated reactions (type I hypersensitivity) is the most common food hypersensitivity in dogs and man and type I, type III, and type IV hypersensitivity are possible immunologic mechanisms in dogs and cats (27). Case 2 showed soft feces and skin problems which are not typical in food hypersensitivity, a few hours after having a snack. The phenotype is very similar to type I hypersensitivity including systemic abnormalities. So far, no report describes ulcerative and split lesions associated with immune-mediated skin diseases with systemic illness induced by foods in dogs.

*Pemphigus* is associated with autoantibodies against desmoglein 1 and desmoglein 3 in the skin junction (31). In humans, *Pemphigus Vulgaris* concurrent with IMHA has been reported (21). In this report, cytokines such as interleukin, tumor necrosis factor-α (TNF-α), and granulocyte-macrophage colony-stimulating factor in blister fluid were measured and TNF-α was significantly increased in the blister fluid (21). Case 3 showed IMHA and it was suspected that those antibodies may be related to antibodies on the red blood cells surface. Based on the hypothesis, the antibodies about desmoglein can attack the red blood cell surface and immune-mediated hemolytic anemia will occur. However, there is no related research.

In the present cases, conventional immune-suppressive drugs (prednisolone and cyclosporine), treatment was well tolerated. However, sometimes additional alternative drugs including hIVIG and chlorambucil were also needed. The mechanism of hIVIG is to block Fc receptors, remove pathogenic autoantibodies, modulate cytokine synthesis, and mediate Fas-Fas ligand interactions (32). In humans, hIVIG is used in thrombocytopenia, lymphocytic leukemia, autoimmune hemolytic anemia, and toxic epidermal necrolysis. However, it is not used well in dermatologic disorders because of side effects (33). In dogs, hIVIG is used in various immune-mediated diseases including IMHA, IMT, *Pemphigus*, and cutaneous drug reactions (32–35).

The present cases were diagnosed as immune-mediated skin diseases concurrent with fatal abnormalities of other organs. The dogs had severe thrombocytopenia, decreased liver function, or secondary IMHA. To the best of the author's knowledge, so far, there are no reports on immune-mediated skin diseases with fatal clinical signs.

## Conclusion

The present case series showed very rare immune-mediated skin diseases concurrent with fatal other organs' abnormalities. A combination of appropriate immune-suppressive drugs with supportive management for the emergency status of systemic dysfunctions yielded successful clinical outcomes in the dogs.

## Data availability statement

The original contributions presented in the study are included in the article/Supplementary material, further inquiries can be directed to the corresponding author.

## Author contributions

SK: clinical management of the first and second cases, writing of the first manuscript draft, and review and editing of the manuscript. YK: clinical management of the third case and writing of the first manuscript draft. H-JK: clinical management of all three cases, review and editing of the manuscript, and study supervision. All authors contributed to write and edit this manuscript.

## Funding

This study was supported by the Basic Science Research Program through the National Research Foundation of Korea (NRF), funded by the Ministry of Education (NRF-2020R1A2C2005364).

## Conflict of interest

The authors declare that the research was conducted in the absence of any commercial or financial relationships that could be construed as a potential conflict of interest.

## Publisher's note

All claims expressed in this article are solely those of the authors and do not necessarily represent those of their affiliated organizations, or those of the publisher, the editors and the reviewers. Any product that may be evaluated in this article, or claim that may be made by its manufacturer, is not guaranteed or endorsed by the publisher.
