# Supplementary material for: Case report: Uncommon immune-mediated skin disease involving systemic disorders in dogs
Source: Front Vet Sci. 2022 Sep 2;9:915775. doi: 10.3389/fvets.2022.915775 (PMC9478576; doi:10.3389/fvets.2022.915775)
Supplement: Supplementary file 1 [file Table_1.pdf]

Supplementary Material

Supplementary table 1. The results of complete blood cell count, serum biochemical profiles, and coagulation test of the three cases

Contents	Results						Reference ranges
	Case 1		Case 2		Case 3		
	Day 0	Day 7	Day 0	Day 10	Day 0	Day 30	
Red blood cells	4.07	5.50	6.07	6.20	1.94	5.34	5.65 - 8.87 M/μL
Hemoglobin	9.0	11.7	14.8	15.1	4.8	12.8	13.1 - 20.5 g/dL
Hematocrit	23.3	31.6	41.4	44.0	15.3	39.0	37.3 - 61.7 %
Reticulocytes	8.1	97.4	31.0	32.9	197.6	42.7	10 - 110 K/ μL
WBC	10.34	16.92	15.12	28.61	36.08	13.69	5.05 - 16.76 K/μL
Neutrophils	0.28	11.19	10.89	22.55	29.85	12.34	2.95 - 11.64 K/μL
Monocytes	5.35	1.34	1.47	1.60	1.38	0.53	0.16 - 1.12 K/μL
Eosinophils	0.45	0.09	0.01	0.01	0.11	0.00	0.06 - 1.23 K/μL
Platelet	107	407	98	173	379	628	148 - 484 K/μL
Total protein	4.9	5.6	7.5	7.6	6.6	7.6	5.2 - 8.2 g/dL
Albumin	1.8	2.3	3.2	3.2	2.7	3.5	2.2 - 3.9 g/dL
Alanine aminotransferase	57	91	68	213	64	15	10 - 100 U/L
Aspartate aminotransferase	22	33	38	60	37	74	0 - 50 U/L
Alkaline phosphatase	212	273	425	484	1021	169	23 - 212 U/L
Gamma-glutamyl transferase	0	2	0	0	36	5	0 - 7 U/L
C-reactive protein	7.8	0.4	>10.0	1.2	>10.0	0.8	0.0 -1.0 mg/dL
aPTT	30.6	-	32.3	-	12.5	-	12 - 28
D-dimer	1988.4	914.94	1961.83	<50.0	901.04	713.00	0 – 250 ng/mL
